# COVID-19 treatments approved in the European Union and clinical recommendations for the management of non-hospitalized and hospitalized patients

**DOI:** 10.1080/07853890.2022.2133162

**Published:** 2022-10-19

**Authors:** Stefania Bellino

**Affiliations:** National Center for Drug Research and Evaluation, National Institute of Health (Istituto Superiore di Sanità), Rome, Italy

**Keywords:** COVID-19 treatments, clinical recommendations, guidelines, European Union

## Abstract

**Background:**

The COVID-19 pandemic caused by SARS-CoV-2 continues to have a serious impact on public health worldwide. Most patients develop mild to moderate symptoms and recover without requiring special treatment, but up to 15% develop severe (dyspnea, hypoxia, lung involvement) or critical symptoms (respiratory failure, septic shock, thromboembolism, multiorgan dysfunction). Although vaccination is having a substantial impact on case numbers, hospitalizations and deaths, there remains a need for new effective treatments against COVID-19.

**Methods:**

This short review aims at reporting on current therapeutics against SARS-CoV-2 focussing on new drugs authorized in the European Union, describing the treatment strategies and the clinical recommendations for the management of hospitalized and non-hospitalized COVID-19 patients based on the available guidelines for clinical practice.

**Results:**

New effective drugs, like antiviral medications and monoclonal antibodies, have been developed as therapy against severe and life-threatening disease courses. Specifically, the European Medicines Agency has authorized two antiviral medicines (nirmatrelvir/ritonavir, remdesivir), supporting also early use of molnupiravir before marketing authorization, and four monoclonal antibodies (regdanvimab, casirivimab/imdevimab, sotrovimab, tixagevimab/cilgavimab). In addition, three drugs (anakinra, tocilizumab, baricitinib) previously authorized for the treatment of rheumatoid arthritis are also available to treat COVID-19.

**Conclusions:**

Recommendations and guidelines for clinical practice should be regularly updated as further evidence becomes available in favour or against specific interventions, to inform all stakeholders involved in the health care of COVID-19 patients both in the community and in the hospital setting, aiming at improving the quality of care and therefore the patient outcome.

**KEY MESSAGES**
- COVID-19 has been recognized as a multisystem disorder affecting many body systems; this wide spectrum of clinical patterns made difficult an appropriate choice of treatments able to counteract severe symptoms of the disease and alleviate the burden on the healthcare system.
- New effective drugs, like antiviral medications and monoclonal antibodies, have been developed and approved by the European Medicines Agency as therapy against severe and life-threatening disease courses.
- Recommendations and guidelines should be regularly updated as further evidence becomes available in favour or against specific interventions aiming at improving the quality of care and therefore the patient outcome.

## 1. Introduction

Since the beginning of the COVID-19 pandemic, lacking specific preventive and therapeutic measures, several pre-existing medicines were used ‘off-label’ with limited scientific evidence for the treatment of SARS-CoV2 infected patients, combining the antiviral activity (i.e. remdesivir, lopinavir/ritonavir, ribavirin), with immunomodulant action (i.e. chloroquine/hydroxychloroquine, azithromycin), and pro-inflammatory cytokines inhibition (i.e. corticosteroids) [1]. Choice of drugs, possible associations, dosages, and duration of treatment was based on the opinion of physicians and previous experience in other similar conditions. Afterwards, several studies were conducted to investigate the real therapeutic effect of these medicines, trying to establish the best pharmacological approach to reduce clinical manifestations, while in the meantime new effective drugs against COVID-19 were developed [2].

This short review aims at reporting on current therapeutics against SARS-CoV-2 focussing on new drugs authorized in the European Union, describing the treatment strategies and the clinical recommendations for the management of hospitalized and non-hospitalized COVID-19 patients.

## 2. Methods

This review was based on data and information retrieved on: (1) COVID-19 treatments authorized for use in the EU; (2) COVID-19 treatments reviewed for use in the EU under Article 5(3) of Regulation 726/2004 to support national decision-making on the possible use of medicines before a formal authorization; (3) medicines marketed for other indications which were made available to patients, even in the absence of a specific therapeutic indication for COVID-19; (4) guidelines on the treatment and management of patients affected by the disease, based on the best available evidence to develop recommendations that guide decisions in public health.

## 3. Results

### 3.1. Covid-19 treatments authorized for use in the European Union

The European Medicines Agency (EMA) has authorized new drugs that may be used for people who have been hospitalized with COVID-19 or for subjects at high risk for developing severe illness, particularly older ages and those with underlying medical conditions such as diabetes, overweight, cardiovascular, kidney, and chronic respiratory diseases, immunosuppression, active cancer, neurodevelopmental disorders [3]. COVID-19 treatments include two antiviral medicines that inhibit viral replication [nirmatrelvir/ritonavir (Paxlovid), remdesivir (Veklury)] and four drugs based on monoclonal antibodies, designed to attach the spike protein of SARS-CoV-2 so that the virus is unable to enter the body’s cells [regdanvimab (Regkirona), casirivimab/imdevimab (Ronapreve), sotrovimab (Xevudy), tixagevimab/cilgavimab (Evusheld)] [2].

Nirmatrelvir/ritonavir is indicated for the treatment of COVID-19 in adults who do not require supplemental oxygen and who are at increased risk for progressing to severe COVID-19 [4]; it should be given as soon as possible after a diagnosis has been made and within 5 days of the start of symptoms to reduce the risk of hospitalization or death. Remdesivir (previously developed against Ebola virus) is indicated for adults and adolescents with pneumonia requiring supplemental oxygen (low- or high-flow oxygen or other non-invasive ventilation at the start of treatment), or in adults who do not require supplemental oxygen and are at increased risk of developing severe illness [5]. Regdanvimab, casirivimab/imdevimab, and sotrovimab are monoclonal antibodies used for treating adults and adolescents who do not require supplemental oxygen and who are at increased risk of progressing to severe COVID-19 [6–8]. Tixagevimab/cilgavimab is the only drug indicated for the pre-exposure prophylaxis of COVID-19 in adults and adolescents, reducing the risk of COVID-19 infection by 77% in the first six months after treatment [9]; tixagevimab/cilgavimab is also indicated for the treatment of individuals with COVID-19 who do not require supplemental oxygen and who are at increased risk of progressing to severe disease.

### 3.2. Treatments not authorized in the European Union specifically for patients with COVID-19

EMA supported national authorities in the EU on the possible early use of molnupiravir (Lagevrio) before marketing authorization, under Article 5(3) of Regulation 726/2004. Molnupiravir can be used for the treatment of COVID-19 in adults who do not require supplemental oxygen and who are at increased risk of progressing to severe disease [10]; it should be administered within 5 days of symptoms onset.

Medicines authorized for another indication in the EU were made available to patients even in the absence of a specific therapeutic indication for COVID-19, and often based on rather limited scientific evidence, particularly in the first phase of epidemic. Specifically, three drugs [(anakinra (Kineret), tocilizumab (RoActemra), and baricitinib (Olumiant)] previously authorized for the treatment of rheumatoid arthritis may also be used in adults with COVID-19 [11–13]. Anakinra is an IL-1 receptor antagonist used in patients with pneumonia requiring supplemental low- or high-flow oxygen at risk of progressing to severe respiratory failure; tocilizumab is an IL-6 receptor antagonist used in patients who are receiving corticosteroids and require extra oxygen or mechanical ventilation; baricitinb is a JAK-inhibitor that can be considered for the treatment of adult subjects hospitalized with severe COVID-19, high-flow oxygen therapy or non-invasive mechanical ventilation, and/or with high levels of systemic inflammation indices.

### 3.3. Therapeutic management of non-hospitalized adults with COVID-19

Different options are available for the treatment of non-hospitalized patients with mild to moderate COVID-19 who are at high risk of disease progression. Symptomatic therapy includes using antipyretics and analgesics (i.e. paracetamol, non-steroidal anti-inflammatory drugs) or antitussives for fever, headache, myalgias, and cough [14–18]. The choice of administering monoclonal antibodies, antivirals, corticosteroids, or anticoagulants is based not only on the patient’s status, but also on other factors which include the clinical efficacy of the product, the feasibility of intravenous injections, the interactions with other concomitant medications, and the prevalence of virus variants. Specifically, nirmatrelvir/ritonavir or molnupiravir can be easily taken orally within 5 days of symptoms onset, however several medicinal products are contraindicated for nirmatrelvir/ritonavir assumption [4]; remdesivir requires intravenous injections starting as soon as possible after a diagnosis of COVID-19 and within 7 days of the onset of symptoms [5]. Concerning monoclonal antibodies, since B.1.1.529 (Omicron) is the dominant SARS-CoV-2 variant in EU, among the monoclonal antibodies a preferential use of tixagevimab/cilgavimab or sotrovimab is recommended, which are active against Omicron, while the others are effective on B.1.1.7 (alpha), and B.1.617.2 (Delta) variants. The use of dexamethasone or systemic corticosteroids is not recommended, but they may be considered in patients who present risk factors for disease progression to severe forms, in the presence of a worsening of pulse oximetry parameters requiring oxygen therapy; however, the total duration of corticosteroids use should not exceed 10 days [14–17]. Moreover, in the initial phase of the disease (with prevailing events related to viral replication) using corticosteroids may have a negative impact on the immune response. The use of low molecular weight heparins in the prophylaxis of thromboembolic events is recommended only in patients with acute respiratory infections and reduced mobility.

Other drugs were used mainly in the first phase of the COVID-19 pandemic based on previous experience in other similar conditions. However, clinical studies showed that chloroquine or hydroxychloroquine with or without azithromycin, lopinavir/ritonavir, and other HIV protease inhibitors should not be administered, and antibiotics should only be considered when the presence of bacterial superinfection is suspected.

### 3.4. Therapeutic management of adults with COVID-19 in the hospital setting

COVID-19 is a complex disease in which respiratory manifestations associated with viral replication are accompanied by systemic effects. The main underlying mechanisms of the disease are an active viral replication followed by a dysregulated immune response characterized by systemic hyperinflammation, including the release of pro-inflammatory cytokines, that leads to organ and tissue damage. Therefore, treatments that reduce the virus replication have a major effect in the early phase of the disease, while anti-inflammatory therapies are more helpful after COVID-19 has progressed to late stages characterized by hypoxia [14]. The use of dexamethasone or other corticosteroids should be considered the standard of care for patients requiring supplemental oxygen with or without mechanical ventilation. Benefits have been demonstrated for remdesivir use in patients with severe but not critical COVID-19, therefore it should only be considered in subjects with pneumonia under oxygen therapy, not requiring high-flow oxygen or mechanical ventilation and with symptoms onset less than 10 days [17–21]. Hospitalized patients with rapidly deteriorating clinical conditions are considered candidates for treatment with tocilizumab or baricitinib which should be given in combination with dexamethasone or another corticosteroid in case of systemic inflammation. Anakinra may be considered for the treatment of hospitalized adults with moderate/severe COVID-19 pneumonia; tocilizumab and baricitinib may be combined in addition to corticosteroids in patients with severe or critical COVID-19.

The use of low molecular weight heparins is recommended in the prophylaxis of thromboembolic events in patients with acute respiratory infections and reduced mobility. Based on clinical studies and clinical experience, chloroquine or hydroxychloroquine, lopinavir/ritonavir, darunavir/ritonavir, and routine use of antibiotics are not recommended in clinical practice.

## 4. Discussion

Although vaccines are the most important public health tool in overcoming the pandemic, effective medicines against SARS-CoV-2 infection are essential to reduce the number of COVID-19 hospitalizations and deaths. COVID-19 has been recognized as a multisystem disorder affecting many body systems; this wide spectrum of clinical patterns made difficult an appropriate choice of treatments able to counteract severe symptoms of the disease and alleviate the burden on the healthcare system. To this aim, different drug classes, including antivirals, immune modulators, anti-inflammatory agents, and anticoagulants have been tested in patients with COVID-19, although disappointing results or no firm conclusions have been drawn in some cases. Indeed, at the early stage of the epidemic, empirical treatments were based on previous experience with the middle east respiratory syndrome coronavirus and on the accumulated experience in managing the disease [22]. Basic therapeutic approaches used for the common cold (i.e. non-steroidal anti-inflammatory drugs, anti-tussive, immune modulators with antibacterial effect) were considered effective in case of mild symptoms and further treatment was not required in the absence of any other clinical manifestations. In the case of pneumonia, it had been recommended to apply the treatment regimen able to prevent cell exposure to the virus and abate the excessive immune reactions (i.e. antiviral agents and immune modulators). In addition to this treatment strategy, anticoagulants could be used.

Currently, antivirals and monoclonal antibodies represent a powerful strategy against COVID-19, even though continuous efforts are needed to explore new potential effective treatments. Antivirals like nirmatrelvir/ritonavir and molnupiravir have the advantage to be available as oral tablets, whereas remdesivir and monoclonal antibodies are not easy to be administered in the outpatient setting, since they have to be given by intravenous infusions. Moreover, monoclonal antibodies are sensitive to viral variants and their efficacy may be reduced. Since most of them neutralized the early strain, Alpha, and Delta variants, the selection of monoclonal antibodies to treat patients who are infected with Omicron variants should be carefully considered. Tixagevimab/cilgavimab retains neutralizing activity against Omicron subvariants, including BA.2.12.1, BA.4, and BA.5, which are currently highly prevalent globally; whereas sotrovimab is active against the Omicron BA.1 and BA.1.1 subvariants, but it has substantially decreased *in vitro* neutralization activity against the Omicron BA.2, BA.4, and BA.5 subvariants [23]. Early initiation of nirmatrelvir/ritonavir, molnupiravir or remdesivir in non-hospitalized patients with mild-to-moderate COVID-19 and risk factors for progression to severe disease has been associated with relative risk reductions in the combined outcome of hospitalization or death, 88% for nirmatrelvir/ritonavir, 30% for molnupiravir, and 87% for remdesivir, respectively [24]. Therefore, in this setting oral nirmatrelvir/ritonavir and intravenous remdesivir seem to be the better choice followed by molnupiravir [25–26]. In addition, initiation of oral antiviral treatments in hospitalized patients (consisting of mostly older people with multiple pre-existing comorbidities) not requiring oxygen therapy showed substantial clinical benefit [27].

## 5. Conclusions

It is deemed important to continuously update the information relating to the efficacy and safety of medicines for COVID-19. Guidelines should be regularly updated as further evidence becomes available in favour or against specific interventions and the use of combination therapy, to inform all stakeholders involved in the health care of patients both in the community and in the hospital setting, aiming at improving quality of care and therefore the patient outcome.

## Author contributions

SB conceived the work, wrote the manuscript, and critically revised the data.

## Data Availability

The data that support the findings of this study are openly available.
